# The Hippo Pathway and Viral Infections

**DOI:** 10.3389/fmicb.2019.03033

**Published:** 2020-01-23

**Authors:** Zhilong Wang, Wanhang Lu, Yiling Zhang, Feng Zou, Zhigang Jin, Tiejun Zhao

**Affiliations:** College of Chemistry and Life Sciences, Zhejiang Normal University, Jinhua, China

**Keywords:** hippo, Yes-associated protein, virus, signaling pathway, pathogenesis

## Abstract

The Hippo signaling pathway is a novel tumor suppressor pathway, initially found in *Drosophila.* Recent studies have discovered that the Hippo signaling pathway plays a critical role in a wide range of biological processes, including organ size control, cell proliferation, cancer development, and virus-induced diseases. In this review, we summarize the current understanding of the biological feature and pathological role of the Hippo pathway, focusing particularly on current findings in the function of the Hippo pathway in virus infection and pathogenesis.

## The Hippo Signaling Pathway

**FIGURE 1:**
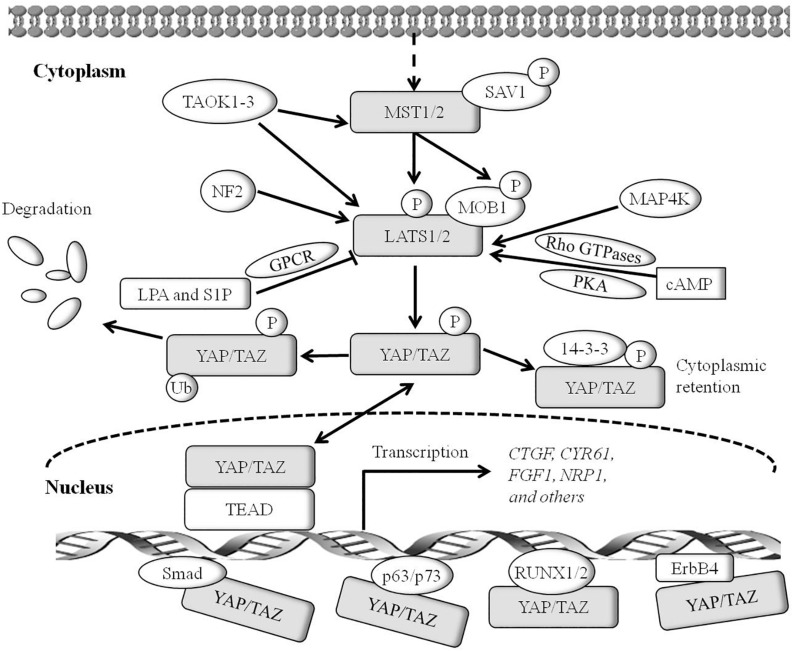
The core Hippo pathway in mammals. Tao kinases 1–3 phosphorylate and activate MST1/2. Interactions between MST1/2 and SAV1 induce LATS1/2 activity. Phosphorylation of MOB1 by MST1/2 enhances MOB1 interactions with LATS1/2, leading to full activation of LATS1/2. NF2 interacts with LATS1/2 and facilitates LATS1/2 phosphorylation by the MST1/2-SAV1 complex. Members of the MAP4K family are identified as direct LATS1/2 activated kinases. cyclic adenosine monophosphate (cAMP) can also activate LATS1/2 kinases *via* protein kinase A (PKA) and Rho GTPases, whereas LPA (lysophosphatidic acid) and S1P (sphingosine 1- phosphophate) are shown to inhibit the activity of LATS1/2 kinases *via* G protein coupled receptor (GPCR). Phosphorylation of YAP on Ser^127^ (TAZ on Ser^89^)induces binding of YAP (TAZ) with 14-3-3, and induces cytoplasmic retention of YAP (TAZ). Phosphorylation of YAP on Ser^381^ (TAZ on Ser^311^) triggers subsequent phosphorylation by casein kinase 1 (CK1*δ*/ε), resulting in the recruitment of SCFβ-TRCP E3 ligase and ubiquitination, and the proteasomal degradation of YAP. TEADs are the major transcriptional activator of YAP in mammals, and YAP binding to TEADs is required for inducing target gene expression. Smad, RUNX1/2, p63/p73, and ErbB4 may be transcriptional factors of YAP/TAZ. YAP and TEADs mediate the expression of target genes such as *CTGF*, *CYR61*, *Birc5*, *FGF1*, *RASSF1A*, and others.

Over the past two decades, numerous studies in *Drosophila* elucidate the central role of the Hippo pathway in organ development (Piccolo et al., 2014). In *Drosophila*, the Hippo pathway, also known as the Salvador-Warts-Hippo pathway and composed of signaling proteins Hpo, Sav, Wts, Mats, and Yki, is a highly conservative signal cascade throughout evolution (Piccolo et al., 2014). Moreover, the Hippo pathway is also highly conserved in mammals, involved in the regulation of cell proliferation, cell contact inhibition, organ size, and tumorigenesis (Zhao et al., 2007). The mammalian orthologs of the Hippo pathway components contain mammalian STE20-like protein kinase 1/2 (MST1/2, also called STK4/STK3; both are Hpo homologs), large tumor suppressor 1/2 (LATS1/2, Wts homolog), Salvador homolog 1 (SAV1, also called WW45), MOB kinase activator (MOB1, Mats homolog), Yes-associated protein (YAP)/transcriptional co-activator with PDZ-binding motif (TAZ, also called WWTR1, both are Yki homologs), and TEA domain-containing sequence-specific transcription factors (TEAD1–4, Scalloped homolog in *Drosophila*) (Figure 1) (Creasy et al., 1996; Lai et al., 2005; Zhang et al., 2008; Zhao et al., 2008a).

**FIGURE 2:**
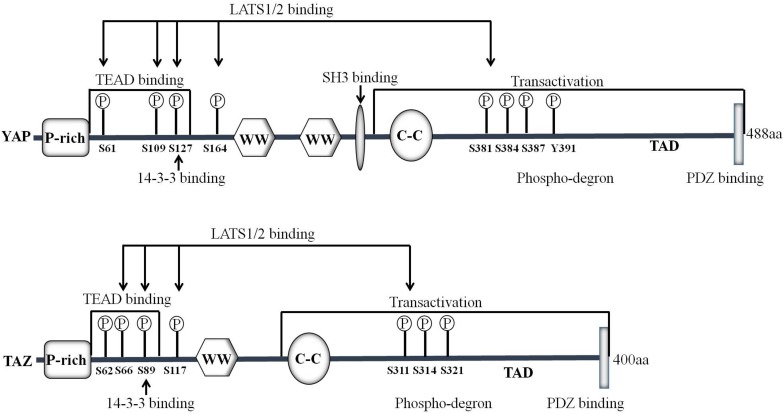
Domain organization and key modifications of YAP/TAZ proteins. YAP is a proline-rich phosphoprotein mainly consisting of the SH3 binding domain, two WW domains and a highly conserved PDZ binding motif FLTWL at the C-terminus. TAZ is homologous to YAP and has only one WW domain. YAP/TAZ is phosphorylated by LATS1/2 on Ser^61^, Ser^109^, Ser^127^, Ser^164^, and Ser^381^ (TAZ Ser^66^, Ser^89^, Ser^117^, and Ser^311^) in the HxRxxS motifs.

In mammals, the Hippo pathway can be initiated by TAOK1–3 kinases, which phosphorylate and subsequently activate MST1/2 (Figure 1) (Boggiano et al., 2011). Evidence shows that MST1/2 activation can be achieved by dimerization and autophosphorylation of MST1/2 (Glantschnig et al., 2002). In association with the regulatory protein SAV1, MST1/2 induces LATS kinase activity by phosphorylating LATS1/2 (Oka et al., 2008). In addition, phosphorylation of MOB1 by MST1/2 enhances the MOB1 interaction with LATS1/2, leading to full activation of LATS1/2 (Ni et al., 2015). Recent reports have found that the neurofibromatosis tumor suppressor NF2 directly binds and recruits LATS1/2 to the plasma membrane, and facilitates LATS1/2 phosphorylation by the MST1/2-SAV1 complex (Yin et al., 2013; Li et al., 2014). In parallel to MST1/2, MAP4K family members have been identified as direct LATS1/2 activating kinases, and cyclic adenosine monophosphate (cAMP) can also activate LATS1/2 kinases *via* protein kinase A (PKA) and Rho GTPases, whereas LPA (lysophosphatidic acid) and S1P (sphingosine1-phosphophate) may inhibit LATS1/2 kinases activity *via* G protein coupled receptor (GPCR) (Yu et al., 2012, 2013; Meng et al., 2015). In addition, TAOK1-3 acts on upstream signaling of MST1/2 to phosphorylate and activate LATS1/2. Activated LATS1/2 directly interacts with and phosphorylates YAP and TAZ. A later study indicates that LATS1 phosphorylates YAP at five sites (Ser^61^, Ser^109^, Ser^127^, Ser^164^, and Ser^381^) in the HxRxxS motifs and TAZ on four HxRxxS motifs (Ser^66^, Ser^89^, Ser^117^, and Ser^311^) (Figure 2) (Zhao et al., 2010a). Mutations in these serine residue sites render YAP/TAZ insensitive to the Hippo pathway. Endogenous YAP/TAZ is localized in both the cytoplasm and nucleus (Hao et al., 2008). Phosphorylated YAP/TAZ will induce their retentions in the cytoplasm and thus cannot regulate the expression of downstream target genes. The two residues most relevant to YAP and TAZ nucleation and degradation are Ser^127^ and Ser^381^ in YAP and Ser^89^ and Ser^311^ in TAZ (Zhao et al., 2010a). Phosphorylation of YAP at Ser^127^ creates a binding consensus for 14-3-3 protein and keeps the YAP in the cytoplasm. Phosphorylation of YAP on Ser^381^ triggers a subsequent phosphorylation by casein kinase 1 (CK1*δ*/ε), resulting in the recruitment of SCF*β*-TRCP E3 ligase, ubiquitination, and proteasomal degradation of YAP (Zhao et al., 2010b). By contrast, when the kinase module is inactivated, hypophosphorylated YAP/TAZ can shuttle to the nucleus where they act as potent transcriptional activators by interacting with the transcription factor TEADs (TEAD 1–4). TEAD is a key transcriptional activator of the Hippo pathway in mammals. YAP is considered as a general transcriptional co-activator for the TEAD transcriptional factors, and it requires binding to TEAD to induce the expression of its target genes such as the connective tissue growth factor (*CTGF*), cysteine-rich angiogenesis inducer 61 (*CYR61*), neuropeptide-1 (*NRP1*) (Zhao et al., 2008b). However, TEAD is not the only DNA binding transcriptional activator with YAP. YAP/TAZ may interact with other transcription factors, including Smad, RUNX1/2, p63/p73, and ErbB4. However, the physiological function of these transcription factors in the Hippo pathway remains unclear (Matallanas et al., 2007; Zhao et al., 2008b). Finally, YAP/TAZ in conjunction with TEADs controls the expression of a wide range of genes that are involved in cell proliferation, differentiation, development, and apoptosis (Figure 1).

Studies in the last decade have demonstrated that YAP/TAZ is a key downstream effector of the Hippo pathway. YAP is first characterized by virtue of its ability to associate with the SH3 (Src homology 3) domain of Yes and Src protein tyrosine kinases (Sudol, 1994). The human *YAP* gene is located on chromosome 11q13, which encodes at least eight YAP protein isoforms (Sudol et al., 1995). There are two major isoforms of YAP: YAP1 containing one WW domain and YAP2 containing two WW domains (Figure 2). The C-terminus of YAP protein contains a highly conserved PDZ-binding motif FLTWL, and this motif is required for YAP nuclear translocation and its regulation of cell cycle and apoptosis (Oka et al., 2010). Through the WW domain, YAP1 forms a functional complex with PPxY motif-containing LATS1 kinase and AMOTL1 protein (Oka et al., 2008; Paramasivam et al., 2011). Yeast two-hybrid screening demonstrates that YAP interacts with the PPxY motif *via* its WW domain for stimulating transcription (Yagi et al., 1999). TAZ is homologous to YAP and has only one WW domain (Figure 2) (Hong and Guan, 2012). TAZ can also play a role in transcriptional co-activation through the combination of WW domain and PPxY motif (Lei et al., 2008). TAZ phosphorylation at Ser^89^ induces the recruitment of 14-3-3, enhances the interactions between TAZ and 14-3-3 and thereby sequestrates TAZ in the cytoplasm (Kanai et al., 2000). It is worth to note that YAP/TAZ mainly functions as a transcriptional co-activator, which regulates the transcription of target genes by translocation between the nucleus and the cytoplasm, thereby affecting cell growth, proliferation, and migration.

## The Pathological Role of YAP/TAZ

Since Hippo pathway activity is important for cell proliferation and regeneration, the Hippo kinase cascade is tightly controlled and regulated in the cell (Plouffe et al., 2015). Consequently, dysregulation of the Hippo pathway can lead to disruptions in cell proliferation, apoptosis, migration, and differentiation and then result in a wide range of diseases including cancers. The genetic evidence in mice indicates that YAP/TAZ plays an important role in developing the normal phenotype of mice, and knockdown of YAP/TAZ in mouse embryonic stem cells results in the loss of OCT4 and SOX2 and consequent differentiation (Varelas, 2014). Moreover, dysregulation of YAP/TAZ may have carcinogen effects because during the development of most cancers, the Hippo pathway may affect the progress of tumorigenesis by regulating the activity of YAP/TAZ (Hong and Guan, 2012). Overexpression and nuclear localization of YAP/TAZ protein have been observed in many human cancers in the liver, esophagus, stomach, prostate, colon, lung, and breast, etc. (Moroishi et al., 2015; Zhang et al., 2015; Kang et al., 2016). Importantly, overexpression of YAP/TAZ correlates with poor prognosis for patients with hepatocellular carcinoma (HCC), colon cancer, esophageal squamous cell carcinoma, late-stage ovarian cancer, non-small-cell lung cancer, and breast tumor (Wang et al., 2010; Chen et al., 2012; Harvey et al., 2013; Xia et al., 2014). During the development of cancers, the regulation of YAP/TAZ by the Hippo pathway is abnormal due to the modulation of the upstream kinases such as LATS1/2 and MST1/2, resulting in hypophosphorylation of YAP/TAZ and its enrichment in the nucleus (Moroishi et al., 2015). Intranuclear retention of YAP/TAZ disrupts the expression of downstream target genes, such as *CTGF*, *CYR61*, *NRP1*, and *FGF1* (Moroishi et al., 2015). Interestingly, recent studies reveal that YAP/TAZ regulates the development of cancer independently of the Hippo-LATS cascade (Moroishi et al., 2015).

Intriguingly, the mammalian Hippo pathway is also associated with non-neoplastic diseases. In some tissues, such as the skeletal muscle and pancreas, disruption of the Hippo pathway is detrimental to the affected tissue, while in other organs such as the heart or brain, loss of the Hippo pathway is beneficial for the injury response and disease delay (Gomez et al., 2014). The Hippo pathway has also been reported to be involved in the development of diseases such as Alzheimer’s disease, polycystic kidney disease, arrhythmogenic heart disease, neurological diseases, and Holt-Oram syndrome (Plouffe et al., 2015). For Alzheimer’s disease, a disease commonly thought to be a neurotoxic disease and caused by the sequential processing of amyloid-β protein precursor (AβPP), TAZ and YAP activate AβPP-mediated signaling of downstream target genes by forming a triple protein complex with the Mint1/Mint3 and AβPP paralogs APLP1/APLP2 (Orcholski et al., 2011).

## Hippo Pathway in Mammalian Immune System

**FIGURE 3:**
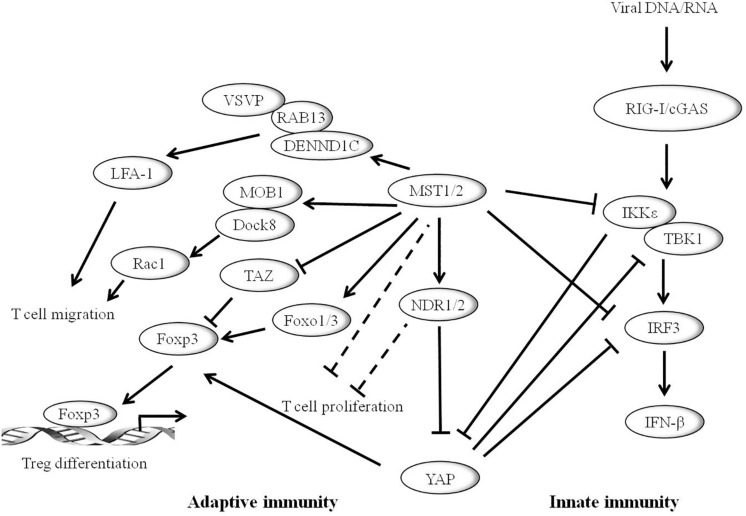
Hippo pathway plays vital role in mammalian immune system. The Hippo core component MST1/2 and YAP can inhibit innate immunity caused by viral infection through the RIG-I/cGAS-TBK1/IKKε-IRF3 axis. IKKε promotes the degradation of YAP to activate cellular antiviral responses. Hippo pathway is also involved in adaptive immunity. MST1/2 enhances T cell migration by activating LFA-1 though DENND1C-RAB13 and VASP signaling, or *via* MST1-MOB1-Dock8-Rac1 axis. MST1/2 promotes regulatory T cell (Treg) differentiation by modulating the Foxp3 expression. YAP promotes the expression of Foxp3, whereas TAZ inhibits its function. Moreover, MST1/2 and/or NDR1/2 may inhibit the development of T cells.

The innate immune system is the first line of defense against microbial infections. Recent studies have shown that YAP/TAZ is a negative regulator of innate immunity against DNA and RNA viruses (Figure 3). YAP may inhibit IRF3 function by direct interactions thereby reducing the production of IFN-β and innate antiviral responses to viruses. After viral infection, YAP deficient mice showed enhanced innate immunity and decreased viral load (Wang S. et al., 2017). In addition, YAP/TAZ abolishes virus-induced activation of TBK1-IKKε by preventing ubiquitination at Lys^63^ in TBK1. Expression of YAP/TAZ impairs virus-driven viral resistance and restores virus replication. Furthermore, knockout of YAP/TAZ or LATS1/2 inhibits antiviral defense and boosts viral replication (Zhang et al., 2017). Moreover, IKKε may phosphorylate YAP at Ser^403^ after viral infection, triggering its lysosomal degradation and resulting in the enhanced cellular antiviral responses (Wang S. et al., 2017). It is well-known that the MST1/2-LATS cascade can negatively regulate the activation of YAP. However, MST1 seems to play a different role in host antiviral defense. MST1 binds directly to IRF3 and phosphorylates its Thr^75^ and Thr^253^ residues, leading to the decrease of IRF3-mediated transcriptional response. Further study reveals that MST1 can inhibit virus-induced TBK1/IKKε activity and restore viral replication (Meng F. et al., 2016). In addition, recent studies show that Toll-like receptor (TLR) signaling can interact with MST1 to regulate host immune responses and promote bacterial killing (White et al., 2019).

A series of studies have connected the Hippo pathway to the regulation of mammalian adaptive immunity (Figure 3). Notably, MST1/2 is critical for T-lymphocyte development, migration, homing, and differentiation. The expression level of MST1/2 in double-positive thymocytes is significantly lower than that of single-positive cells. Loss of MST1/2 results in decreases in the number of peripheral CD4^+^ and CD8^+^ T cells. Thus, it is suggested that MST1/2 is critical for the later stage of T cell development (Mou et al., 2012). MST1/2 may regulate CD4^+^ T-cell activation and proliferation through MOB1A/B and/or NDR1/2 (Cornils et al., 2010). In addition, MST1/2 enhances T cell migration by activating LFA-1 through DENND1C-RAB13 and VASP signaling, or *via* MST1-MOB1-Dock8-Rac1 axis, finally affecting T-cell immune response (Hong et al., 2018). Accumulating evidence shows that the Hippo pathway has emerging roles in effector T cell differentiation. MST1/2 promotes Foxp3 expression and regulatory T cell (Treg) differentiation by regulating the stability of forkhead box O1/3 (Foxo1/3) (Du et al., 2014). A recent study shows that TAZ attenuates the acetylation of Foxp3 and inhibits Treg differentiation, while TAZ binds to ROR*γ*t (RAR related orphan receptor gamma t) and promotes Th17 differentiation. Unlike TAZ, YAP up-regulates Foxp3 expression and enhances TGF*β*/Smad pathway and Treg function (Hong et al., 2018).

**FIGURE 4:**
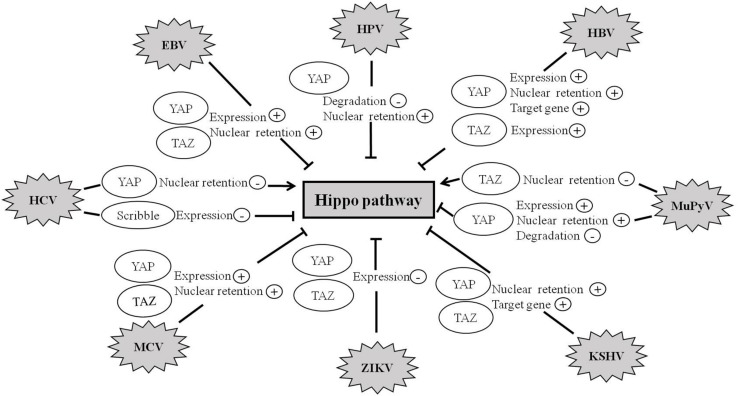
Hippo pathway is involved in viral infection. Abnormal regulation of the Hippo pathway has been observed during infection with a variety of viruses such as HBV, HCV, MCV, ZIKV, EBV, KSHV, HPV, and MuPyV. Viruses exert their carcinogenic function by regulating the expression, degradation, and nuclear retention of YAP/TAZ proteins.

Recent studies further suggest that the Hippo pathway also plays crucial roles in virus-induced diseases. To date, a variety of human viruses exert their carcinogenic function by activating the activity of YAP and TAZ (Figure 4 and Table 1).

**TABLE 1:** Functions of the Hippo pathway in viral infections.

**Virus**	**Functions and characteristics**	**References**
Hepatitis B virus (HBV)	HBx binds to the promoter of YAP and activates it through CREB; HBx up-regulates YAP expression through down-regulating miRNA-375.	Zhang et al., 2012
	YAP expression is significantly up-regulated, and YAP located in the nucleus of HBV-infected hepatoma cell lines.	Wu et al., 2016
	HBXIP up-regulates YAP through activating the transcription factor c-Myb in HCC cells.	Wang Y. et al., 2017
	HBV encoded preS2 protein promotes the expression of TAZ at the protein level by repressing miRNA-338-3p.	Liu P. et al., 2015
	The YAP-target genes were up-regulated in the Alb-preΔS2 mice.	Teng et al., 2017
Hepatitis C virus (HCV)	HCV E2 protein mimics Glypican3 (GPC3) and interacted with CD81, leading to decrease the expression of YAP in nuclear.	Xue et al., 2018
	HCV NS4B induces EMT by inhibiting the Hippo pathway.	Hu et al., 2017
Molluscum contagiosum virus (MCV)	After MCV infection, the expression of YAP and TAZ in the peripheral keratinocytes was significantly increased.	Seo et al., 2018
Human papillomavirus (HPV)	HPV E6 protein maintains YAP protein levels by inhibiting proteasome-dependent protein degradation; knockout of YAP prevents HPV16 E6 from inducing cell proliferation.	He et al., 2015
	HPV E6 can regulate YAP nuclear localization by interacting with cellular PDZ domain proteins.	Webb Strickland et al., 2018
	YAP expression pattern exactly matches HPV DNA distribution in the HPV16-containing organotypic HFK raft culture system.	Xiao et al., 2014; He et al., 2015
	YAP protein was accumulate in the nucleus of HPV-OPSCCs.	Alzahrani et al., 2017
	Activation of YAP1 in the cervical epithelium leads to congenital antiviral immunodeficiency, causing persistent HPV infection.	He et al., 2019
Murine polyomavirus (MuPyV)	PyST reduces YAP phosphorylation, leading to elevated levels of YAP protein and its nuclear localization; PyST inhibits the binding of YAP and βTRCP; YAP and PyST interaction is necessary for PyST to repress myoblast differentiation and to induce IBMX-dependent apoptosis.	Hwang et al., 2014
	PyMT binds to PP2A on the cell membrane, resulting in phosphorylation of PyMT.	van der Meijden and Feltkamp, 2018
	YAP/MT interaction allows it to bind to PP2A, resulting in stabilization of YAP protein and its membrane localization.	Rouleau et al., 2016
	PyMT activates LATS in a Src-dependent manner, which leads to phosphorylation and cytoplasmic accumulation of TAZ protein.	Shanzer et al., 2015
Kaposi sarcoma-associated herpesvirus (KSHV)	KSHV vGPCR protein acts through Gq/11 and G12/13 to promote YAP/TAZ dephosphorylation and nuclear translocation, resulting in transcriptional activation of YAP/TAZ target genes.	Liu G. et al., 2015
Epstein–Barr virus (EBV)	STK4 deficiency is very susceptible to EBV infection, leading to lymphoid hyperplasia and lymphoma development.	Schipp et al., 2018
	In EBV-positive gastric cancer, miR-375, which exerts tumor suppressor function through down-regulating YAP1, TEAD4, and CTGF, was down-regulated.	Kang et al., 2018
	EBV-encoded latent membrane protein LMP1 promotes TAZ protein expression and nuclear translocation.	He et al., 2017
Zika virus (ZIKV)	ZIKV infection induces hypermethylation of TAZ and RASSF1; YAP/TAZ and phosphorylated YAP/TAZ levels are significantly reduced in hNPCs after ZIKV infection.	Kandilya et al., 2019

## Hepatitis B Virus

Hepatitis B virus (HBV) infection is one of the main causes of hepatocellular carcinogenesis (El-Serag and Kanwal, 2014). Among HBV-encoded viral proteins, HBV X (HBx) is a multifunctional regulatory protein and plays a crucial role in HCC (Tang et al., 2006). Recent research has shown that compared with normal controls, the expression level of YAP is remarkably elevated in HCC samples and HBV-infected hepatoma cell lines (Wu et al., 2016). YAP expression is positively correlated with HBx expression in HBV-positive HCC tumor tissues. In cell lines, the level of YAP is significantly up-regulated in liver tissues of HBx transgenic mice. Immunohistochemistry (IHC) studies indicate that YAP is mainly located in the nucleus of HBV-positive HCC cells (Wu et al., 2016). It is found that HBx could bind to the promoter of YAP and activate it through the CREB protein. Moreover, knockdown of YAP can significantly block HBx-induced proliferation of hepatoma cells *in vivo* and *in vitro* (Zhang et al., 2012). Further study reveals another mechanism to increase YAP expression by HBx, in which HBx up-regulates YAP expression *via* down-regulation of miRNA-375 (Zhang et al., 2012). In addition, Wang Y. et al. (2017) reported that hepatitis B X-interacting protein (HBXIP) is able to up-regulate YAP expression in hepatoma cells. A further study demonstrates that HBXIP may up-regulate YAP through activating the transcription factor c-Myb in HCC cells. Silencing of YAP abolishes the proliferation of hepatoma cells mediated by HBXIP *in vitro* and *in vivo*. It thus indicates that HBXIP contributes to the growth of hepatoma cells through the YAP protein (Wang Y. et al., 2017).

Besides HBx, HBV-encoded preS2 protein also contributes to the development of HCC. preS2 may promote the expression of TAZ at the protein level but not at the mRNA level by repressing miRNA-338-3p (Liu P. et al., 2015). Moreover, knockdown of TAZ impairs preS2-promoted HCC proliferation and migration, indicating that TAZ is needed for preS2-induced HCC (Liu P. et al., 2015).

Pre-S deletion of the HBV surface antigen is prevalent in HBV carriers, and the presence of pre-S2 deletion mutants is highly associated with cirrhosis and HCC. After a 30-month follow-up observation of Alb-preΔS2 transgenic mice, it shows that the YAP-target genes, such as *Birc5*, *Ankrd1*, *CTGF*, and *CYR61*, are aberrantly up-regulated in Alb-preΔS2 mice (Teng et al., 2017). Thus, these findings suggest that the Hippo core component YAP may be activated in Alb-preΔS2 transgenic mice, and the Hippo pathway may be involved in the HBV-induced hepatocarcinogenesis and pathogenesis of liver diseases. Taken together, these data reveal that the Hippo pathway may play a key role in the development of HBV-induced diseases.

## Hepatitis C Virus

Hepatitis C virus (HCV) infection causes acute and chronic hepatitis and can lead to permanent liver damage and HCC. The initial study by Xue et al. (2018) found that Glypican3 (GPC3), a glycosylphospatidyl inositol-anchored membrane protein, is overexpressed in HCC in contrast to the trivial expression in normal hepatocytes. The elevation of GPC3, by interacting with CD81, enhances the Hippo pathway and decreases the phosphorylation of the protein Ezrin, the amount of YAP in the nuclear, and cell proliferation (Xue et al., 2018). Further study demonstrates that the activation of CD81 by binding with agonistic antibody may suppress the Hippo pathway, leading to the nuclear translocation of YAP. Moreover, the E2 protein of HCV mimics GPC3 and promotes the development of HCCs *via* the stimulation of the Hippo pathway in hepatocytes (Xue et al., 2018).

It is well-known that HCV non-structural protein NS4B plays a critical role in HCV life cycle and contributes to carcinogenesis. However, the role of NS4B in the development of epithelial–mesenchymal transition (EMT) remains poorly understood. A recent study indicates that overexpression of NS4B inhibits the expression of Scribble protein and subsequently inactivates the Hippo pathway, resulting in the activation of the PI3K/AKT pathway and up-regulation of Snail. Given that Snail is associated with EMT, it is thus likely that Scribble-Hippo-PI3K/AKT pathway may be involved in HCV induced EMT (Hu et al., 2017). Understanding the complex relationship between HCV and the Hippo signaling may provide a novel direction for discovering new treatments for patients with the liver cancer.

## Molluscum Contagiosum Virus

Molluscum contagiosum virus (MCV) is the sole human poxvirus of the *Molluscipoxvirus* genus, and its infection can induce molluscum contagiosum (MC) (Shisler, 2015). Although it has been reported that MCV may inhibit innate immune responses, the underlying mechanism remains unclear. In the samples of MCV-infected patients, the expression of YAP and TAZ in the nucleus and cytoplasm of peripheral keratinocytes is significantly increased, compared to that of normal subjects (Seo et al., 2018). However, the expression of YAP in intracytoplasmic inclusions is markedly decreased in the upper level of the epidermis (Seo et al., 2018). The mRNA levels of *YAP* and *TAZ* in MC lesions are significantly increased compared to that of normal lesions (Seo et al., 2018).

Recent studies indicate that the MCV-encoded MC160 protein may inhibit TBK-1 and IRF3 activation (Randall et al., 2014; Biswas et al., 2018). It is well-known that YAP/TAZ acts as a natural inhibitor of TANK binding kinase 1 (TBK1), which is a major modulator for sensing cytosolic nucleic acid and regulating antiviral defense (Wang S. et al., 2017; Zhang et al., 2017). Loss or inactivation of YAP/TAZ may relieve suppression on TBK1 thus boosting antiviral responses.

Therefore, MCV may contribute to the development of MC *via* increasing the expression of YAP and TAZ and the suppression of TBK1.

## Human Papillomavirus

Human papillomavirus (HPV) infection is a major risk factor for cervical cancer (He et al., 2015). HPV encodes two major oncoproteins, E6 and E7, which are required for the continued proliferation of tumor-derived cell lines (He et al., 2015). He et al. (2015) reported that YAP is highly expressed during cervical cancer progression, leading to the proliferation and migration of cervical cancer cells. In cervical cancer, YAP stimulates the expressions of TGF-*α* and AREG, which in turn activates EGFR, leading to suppression of both the Hippo pathway and subsequent activation of YAP (He et al., 2015). Thus, the TGF-*α*/EGFR pathway interacts with the Hippo/YAP signaling to form a loop, which may play a critical role in regulating cervical cancer progression. After HPV infection, the E6 protein decreases the expression of p-YAP (S397) and maintains the level of YAP by preventing its degradation from proteasome-dependent pathways (He et al., 2015). Moreover, knockdown of YAP may impair HPV16 E6-induced cell proliferation, indicating that YAP may be involved in HPV E6-mediated regulation on the growth of cervical cancer cells (He et al., 2015).

The most recent study has shown that HPV E6 can regulate the nuclear localization of YAP by interacting with cellular PDZ domain proteins, including LRPPRC, RLGAPB, EIF3A, SMC2/3, AMOT, AMOTL1, and ARHGEF1 (Webb Strickland et al., 2018). Interestingly, YAP expression pattern exactly matches HPV DNA distribution in the HPV16-containing organotypic HFK raft culture system. These studies identify that the E6 activity is associated with cell transformation (Xiao et al., 2014; He et al., 2015).

Scribble has been confirmed as an important regulator for the Hippo signaling cascade and has recently been found to be involved in the development of cervical cancer through HPV E6 oncoprotein. Alzahrani et al. (2017) reported that the Scribble interacting protein nitric oxide synthase 1 adaptor protein (NOS1AP) forms a complex with YAP. Importantly, a further study indicates that the Hippo pathway is inhibited in HPV-infected tumors while YAP protein is significantly accumulated in the nucleus of HPV-oropharyngeal squamous cell carcinoma (HPV-OPSCC) cells (Alzahrani et al., 2017). These data suggest that the Hippo pathway may be important for causing OPSCC.

An unconventional mechanism of cervical cancer caused by HPV infection is discovered in a recent report: He et al. (2019) by studying the YAP1 activity in transgenic mice found that hyperactivation of YAP1 in cervical epithelial cells facilitated HPV infection *via* increasing the putative HPV receptors. HPV synergizes with hyperactivated YAP to promote the development of cervical cancer. Furthermore, the expression and activation of key components of the type I IFN pathway are suppressed by the expression of YAPS127A, which is a constitutively active YAP1 mutant. The inhibition of innate immunity by YAP is further confirmed in the K14-YAPS127A transgenic mouse (He et al., 2019). Therefore, this study indicates that the activation of YAP1 in cervical epithelial cells may result in defective innate antiviral immunity, which may allow HPV to escape immune surveillance, leading to persistent HPV infection (He et al., 2019). Consistently, a recent finding shows that YAP may reduce innate antiviral immunity by suppressing TBK1 activity (Zhang et al., 2017).

In summary, these studies suggest that the Hippo/YAP1 pathway significantly contributes to cervical carcinogenesis partly due to HPV-induced activation of YAP1 that acts on both the initiation and progression of cervical cancer.

## Murine Polyomavirus

Murine polyomavirus (MuPyV) is a small DNA virus that often induces tumors in newborn animals (Heiser et al., 2016). Three early gene products of MuPyV include large T (PyLT), middle T (PyMT), and small T (PyST). Among them, PyST is a viral oncogene that regulates cell cycle, cell survival, apoptosis, and differentiation (Hwang et al., 2014). Recent evidence suggests that PyST reduces YAP phosphorylation by bringing together protein phosphatase 2A (PP2A) and YAP protein, resulting in an increase in the YAP expression and nuclear localization (Hwang et al., 2014). Ubiquitin ligase β-transduction protein repeat protein (βTRCP) is known to interact with YAP for degrading YAP in a phosphorylation-dependent manner. Moreover, PyST greatly attenuates the combination of YAP and βTRCP (Hwang et al., 2014). Genetic analysis reveals that YAP binding is needed for PyST to repress myoblast differentiation but also to induce isobutylmethylxanthine (IBMX)-dependent apoptosis in 3T3-L1 preadipocytes (Hwang et al., 2014).

Of note, PyMT in human polyomavirus may bind to PP2A on the cell membrane and interact with Src tyrosine kinase, which results in the phosphorylation of PyMT and regulation of related cell signaling pathways (van der Meijden and Feltkamp, 2018). Rouleau et al. (2016) reported that YAP binding to PyMT is important for PyMT-mediated tumor transformation. In addition, binding of YAP to MT brought it together with PP2A, leading to dephosphorylation of YAP in this triple complex (Rouleau et al., 2016). It finally led to the stabilization of YAP protein and induced YAP localized to the membranes (Rouleau et al., 2016). Interestingly, TAZ, the YAP paralog, is required for PyMT-induced cell transformation. The latest study has shown that PyMT activates the tumor suppressor LATS of the Hippo pathway in a Src-dependent manner (Shanzer et al., 2015). This process may enhance phosphorylation and cytoplasmic accumulation of TAZ proteins and also nuclear exclusion of Shp2, which all aid PyMT-induced cell transformation.

## Kaposi Sarcoma-Associated Herpesvirus

Kaposi sarcoma (KS) is a malignant tumor caused by Kaposi sarcoma-associated herpesvirus (KSHV) infection (Purushothaman et al., 2016). Among the KSHV-encoded genes, viral G-protein coupled receptor (vGPCR) has been proposed to play important roles in KSHV induced angiogenesis, while the precise molecular mechanism of how vGPCR participates in KSHV-induced oncogenesis is still unclear (Liu G. et al., 2015). The initial study by Liu G. et al. (2015) indicates that YAP and TAZ expression in KS tumors is significantly higher than that in normal tissues, suggesting that KSHV infection may lead to great activation of both YAP and TAZ. Furthermore, KSHV-encoded vGPCR protein acts through Gq/11 and G12/13 to promote YAP and TAZ dephosphorylation and nuclear translocation, resulting in transcriptional activation of YAP/TAZ target genes. Conversely, silencing of YAP and TAZ may inhibit vGPCR-mediated cell proliferation and tumorigenesis (Liu G. et al., 2015). These data collectively indicate that YAP and TAZ activation is important for vGPCR-induced tumorigenesis.

## Epstein–Barr Virus

Epstein–Barr virus (EBV) is a member of the herpesvirus family that can cause many human malignancies, such as nasopharyngeal carcinoma, Burkitt’s lymphoma, T-cell lymphoma, gastric carcinoma, and breast cancer (Liu et al., 2006; Holmes, 2014). STK4/MST1 is the central component of the Hippo pathway that controls cell growth and apoptosis (Abdollahpour et al., 2012). Defects in human STK4 cause primary immunodeficiency syndrome reducing T cell and B cell (Zhou et al., 2008; Abdollahpour et al., 2012). Case reports indicate that many patients with STK4 deficiency are highly susceptible to EBV infection, leading to lymphoid hyperplasia and lymphoma development (Schipp et al., 2018). Therefore, STK4 is critical for the control of unrestricted EBV-induced lymphoproliferation.

Gastric cancer (GC) is a complicated and heterogeneous disease, attracting global health concern (Uemura et al., 2001). GC is characterized by four distinct molecular subtypes: EBV-positive, chromosomal instability (CIN), microsatellite instability (MSI), and genomically stable (GS) (Cancer Genome Atlas Research Network, 2014). It is reported that miR-375 exerts tumor suppressor function in GC through down-regulating the expression of three components of the Hippo pathway, YAP1, TEAD4, and CTGF (Kang et al., 2018). Notably, miR-375 is significantly down-regulated in EBV-positive GC subtypes due to promoter methylation (Kang et al., 2018). It is thus likely that Hippo/YAP1 pathway may participate in the development of EBV-positive GC subtypes through silencing of miR-375 expression.

The EBV-encoded latent membrane protein, LMP1, is an essential oncogenic protein in EBV-induced growth transformation of human B lymphocytes (Kulwichit et al., 1998). A recent study reveals that LMP1 promotes the expression of TAZ in nasopharyngeal carcinoma cells (He et al., 2017). LMP1 increases the level of TAZ protein not by inducing its transcription but by increasing protein synthesis and stabilization (He et al., 2017). In addition, EBV-LMP1 inhibits phosphorylation of LATS1/2 by interacting with gelsolin, thereby stabilizing TAZ and promoting its nuclear translocation. Finally, the knockdown experiment demonstrates that TAZ is critical for LMP1-induced cell proliferation, EMT, and cancer stem cell-like properties in nasopharyngeal carcinoma (He et al., 2017). These findings provide new insights into the carcinogenic effect of the Hippo pathway in EBV-mediated oncogenesis.

## Zika Virus

Zika virus (ZIKV) is a re-emergence of mosquito-borne flavivirus that usually causes mild symptoms in patients (Dai et al., 2016). Extensive studies indicate that ZIKV infection is associated with neonatal microcephaly. DNA methylation profiling reveals that ZIKV infection induces hypermethylation on genes of key signaling molecules in the Hippo signaling pathway, such as TAZ and RASSF1, resulting in a decrease in the protein expression. In addition, the expression level and phosphorylation of YAP/TAZ are significantly decreased in human neural progenitor cells (hNPCs) after ZIKV infection (Kandilya et al., 2019). These data suggest that ZIKV may inhibit cell proliferation by down-regulating the Hippo signaling.

## Perspective

This review outlines the importance of the Hippo/YAP pathway in the control of organ size, cell proliferation, cell growth, tumorigenesis, and virus-induced diseases. The correlation between the Hippo pathway and virus-induced diseases gradually attracts great attentions from the scientific community, whereas there are many key issues that remain to be addressed. Although the Hippo pathway is involved in the development of many diseases caused by viruses, it is of interest whether virus-induced diseases can be ameliorated by modulating the Hippo signaling pathway. For instance, LATS1/2 kinase can effectively reduce the inhibition of TBK1/IKKε by YAP/TAZ, suggesting that YAP/TAZ-related antiviral responses might be controllable. However, the underlying molecular mechanism requires further investigation (Zhang et al., 2017). In addition, YAP negatively regulates the antiviral natural immune response and YAP deficiency leads to the enhancement of natural immunity (Wang S. et al., 2017). While the Hippo pathway is off, the YAP/TAZ driving function mainly enters the nucleus and binds to transcription factors of the TEAD family to induce gene transcription (Meng Z. et al., 2016). Drug manipulation of the components of the Hippo pathway may provide a potential value for antiviral prophylaxis. Verteporfin (VP) and vestigial-like family member 4 (VGLL4) are found to have the function of eliminating the interaction between YAP and TEAD (Zhang et al., 2014; Feng et al., 2016). Statins, also known as HMG-CoA reductase inhibitors, can retain YAP and TAZ in the cytoplasm, thereby inhibiting YAP-induced transcription (Sorrentino et al., 2014). Therefore, future studies focusing on the exact role of YAP/TAZ in response to viral infection will provide insights into pathogenesis and are necessary for the development of potential therapeutic strategies targeting YAP/TAZ in a variety of virus-induced diseases.

## Author Contributions

All authors listed have made a substantial, direct and intellectual contribution to the work, and approved it for publication.

## Conflict of Interest

The authors declare that the research was conducted in the absence of any commercial or financial relationships that could be construed as a potential conflict of interest.
